# Why did the Japanese Government take so long to approve the intrauterine contraceptive device?

**DOI:** 10.1016/j.rbms.2018.09.002

**Published:** 2018-10-16

**Authors:** Aya Homei

**Affiliations:** School of Arts, Languages and Cultures, The University of Manchester, Manchester, UK

**Keywords:** intrauterine device (IUD), Ōta ring, birth control, post-war, Japan, transnational population control movement

## Abstract

While the majority of East Asian countries embraced the modern intrauterine device (IUD) during the 1960s, the sale and distribution of the IUD in Japan was not authorized until 1974. In this paper, I address why the Japanese Government took so long to permit the use of the IUD. Firstly, I examine scientific debates in Japan during the early 1950s on the efficacy of the IUD and associated health risks, to illustrate how the Government's conservative attitude was fostered by a co-constitutive relationship between health officials and leading obstetrician-gynaecologists who believed that the IUD was dangerous and likely to induce abortion. I also trace the Japanese Government's rapidly changing attitude through the 1960s, and analyse the influential interaction between national policy making and the enthusiastic response of a small number of Japanese doctors to the transnational movement to curb population growth in developing countries. I argue that the specific ways in which biomedical discourse was shaped by the sociopolitical position of doctors in relation to the Government's health administration explains the Japanese Government's resistance to use of the IUD. However, I also note that the Government's dramatic change in attitude was influenced directly by transnational reproductive politics. This paper will enhance the history of reproductive politics in post-war Japan, which has tended to focus on the politics surrounding abortion and the contraceptive pill.

## Introduction

Since its inception in the 1960s, the modern form of the intrauterine device (IUD) has been one of the most commonly used contraceptives in the world (d’Arcangues, 2007, United Nations Department of Economic and Social Affairs Population Division, 2011), particularly in East Asia where the method has been consistently popular (d’Arcangues, 2007, Donaldson, 1990, Lethbridge and Wang, 1991). However, use of the IUD in Japan remained low for most of the post-war period (Tama, 2016: 128), and even today, the IUD is less popular than the contraceptive pill, another method generally avoided (Nakamura and Kitamura, n.d, Ministry of Health, Labour and Welfare, 2011). The low uptake of the IUD in Japan is both striking and unique.

The reasons behind this are multifaceted, but the Japanese Government's longstanding reluctance to approve use of the IUD provides a significant clue to understanding the current position. Commercial production and distribution of IUD contraceptives was only authorized in 1974. This is a remarkable fact when we consider the following three points. First, the Japanese physician, Tenrei Ōta, became internationally renowned for inventing a precursor of the modern IUD named the ‘Ōta ring’ (‘Ōta ringu’; hereafter, ‘the ring’). Despite this device being available in Japan from the early 1930s, it would be over 40 years before the Japanese Government officially recognized this contraceptive method as a legitimate form of birth control. Second, due to anxieties concerning over-population in the early post-war period, the Japanese Government was keen to promote birth control, and implemented a state policy to this effect in 1951. Despite this incentive, the Government did not sanction the Ōta ring – then being used in clinical trials – when licensing medical contraceptives. Finally, as indicated above, the governments of neighbouring Taiwan, South Korea and the People's Republic of China embraced the IUD enthusiastically during the 1960s, regarding it as an effective tool for the fertility reduction programmes in these countries. In this context, why did the Japanese Government remain practically impervious to events in the region, and take so long to authorize the commercial production and distribution of IUD contraceptives?

In this paper, I engage with this question by examining how doctors working with the IUD interacted with policy makers in Japan during the post-war period. As Norgren and Steslicke have shown, doctors in post-war Japan, both as individuals and as a collective, had a great deal of leverage in shaping policies that had a direct impact on the reproductive lives of ordinary people (Norgren, 2001, Steslicke, 1973). Primarily motivated to protect their lucrative private practices, doctors exerted most influence via electoral politics, constituting a powerful ‘pressure group’ (‘atsuryoku dantai’). However, historical research has tended to focus primarily on doctors as political figures, neglecting the interaction between their scientific profile and political role. As I will demonstrate, how the intersection between science and policy was co-produced with the social order was as significant for reproductive politics in modern Japan as anywhere else (e.g. Greenhalgh, 2005, Greenhalgh, 2008). How did the practice of science interact with the political role of doctors, and how did this interaction manifest in the Japenese Government's approach to the IUD? These are additional questions I will address in this paper.

Firstly, I examine the Japanese Government's reluctance to authorize the IUD during the 1950s, in what became known as the ‘ring dispute’; a debate between clinical and academic doctors over the validity of the contraceptive for fertility regulation. While clinical doctors stressed the ring's ability to limit family size, leading academic doctors rejected this view and emphasized the contraceptive's potential health risks. The result of the debate was determined by relative proximity to political power; the anti-ring faction was closer to the decision-making process. I argue that the Japanese Government's conservative attitude to the contraceptive owed much to the advantageous position of anti-ring doctors, and the opportunity this afforded them to influence the trajectory of birth control policy.

While the first part of this paper focuses on internal factors, the second part looks beyond the national framework. As recent work has shown, from the 1960s onwards, the IUD was promoted globally via transnational networks mobilizing family planning initiatives to reduce fertility rates as part of development programmes in Asia, Africa and Latin America (Connelly, 2008, Donaldson, 1990, McCann, 2016: 143–152; Takeshita, 2011). The process leading to official approval of the IUD in Japan was directly influenced by this transnational trend. As I will show, Japanese doctors participated in scientific activities to further transnational IUD circulation, and this participation would ultimately sway official attitudes to the IUD in Japan during the 1960s.

Birth control in Japan's modern history – taken in a broad sense, and therefore including abortion – has attracted substantial academic attention over the past few decades (Callahan, 2004, Coleman, 1991, Drixler, 2013, Fujime, 1997, Gordon, 2005, Hopper, 1996, Kato, 2009, Norgren, 2001, Obayashi, 2006, Ogino, 2008b, Ōta, 1976, Sawada, 2014, Suzuki, 2002, Tama, 2001, Tama, 2006, Tama, 2014, Tama, 2016, Tipton, 1994, Tsukamoto, 2014). This paper builds on this body of work in three significant ways. First, as mentioned above, in contrast to earlier work that primarily studied doctors as political interest groups, this paper focuses on the nexus between the scientific profile of medical doctors, their social status and the Japanese Government's stance on birth control, by drawing on work that highlights the co-constitutive relationship between science and sociopolitical order (e.g. Jasanoff, 2006). This perspective challenges existing work on the history of reproductive politics in Japan, which mainly focuses on sociopolitical factors but overlooks the politics engrained in scientific activities.

Second, by focusing on the IUD, this paper also departs from the existing English language history of birth control in post-war Japan, which has centred on the predominance of abortion and lack of interest in the contraceptive pill. This dominant narrative can lodge our perspective too tightly within the domestic framework, and consequently obscure the element of transnationalism that significantly influenced politics at the national level. This IUD case study illuminates the transnational dimension in the domestic policy-making process, and invites a reappraisal of how the state should be presented in historical discussions of Japanese reproductive politics.

With this perspective, the paper first provides context by introducing the development of the IUD in pre-war and wartime Japan, and the adoption of the device by birth control activists immediately after the war. These sections will be followed by a discussion of the 1950s ‘ring dispute’ and the emerging factors that contributed to the Japanese Government's negative attitude towards the contraceptive. The final section will depict the rise of the transnational population-control movement, and describe how the engagement of Japanese doctors with this movement coincided with a shift in the Government's approach to the IUD, eventually leading to official approval of the contraceptive in 1974.

Although my focus is the process towards governmental authorization of the IUD in Japan, I in no way advocate the progressivist view that the IUD was inherently a cutting-edge technology, and therefore should have gained approval earlier than 1974. Rather, my approach is similar to science, technology and society scholars such as Takeshita, who examined IUD development in the USA, and concluded that the technology was ‘politically versatile’ (Takeshita, 2011: 3), shaped by a politically charged discursive force and, at times, conditional to historical, social and scientific arrangements. This understanding enables us to focus on multiple contingent factors, and thus interpret the Japanese Government's resistance to the contraceptive in a way that goes beyond a progressivist framework.

## The IUD in pre-war and wartime Japan

While an internalist account of the history of IUD contraceptives identifies the Berlin-based gynaecologist Ernst Gräfenberg as the chief pioneering figure, it simultaneously describes Ōta as another early inventor (Thiery, 1997, Thomsen, 1982, Tietze, 1995).

Ōta was from a medical family with a strong gynaecological tradition. However, according to Ōta himself, it was sheer curiosity, rather than family tradition or financial gain, which motivated him to specialize in birth control (Ōta, 1979: 237). Inspired by the ‘traditional tale from East Asia’ that claimed a cow would not get pregnant if a golden ball was inserted into its uterus (Ōta, 1979, 237), Ōta worked on intrauterine contraceptive methods through the 1920s. Having learned about the Gräfenberg ring in 1931, Ota decided to develop his own. He created a coil made of gold with a golden ball attached, and, mirroring Gräfenberg, named the device the ‘Ōta ring’. Having conducted a small-scale trial with relatives and acquaintances, Ota published his results in 1932 (Ōta, 1932).

The Ōta ring did not gain popularity in the 1930s. Although there were various reasons for this, the negative attitude to birth control prevalent within Japanese officialdom certainly played a critical role. The birth control movement had been gaining popularity in Japan's cities during the 1920s, but associations with socialism and labour organizations led to concerns within the Japanese Government that its authority was being subverted, and the movement's activities were restricted (Fujime, 1997, Obayashi, 2012, Takeuchi-Demirci, 2010). Ōta himself became a victim of government suppression due to his cooperation with social activists. In this political climate, the Ōta ring, invented and distributed by an individual deemed politically suspect, had limited reach among the public.

Furthermore, the small group of doctors that had political influence during the 1930s were reluctant to publicly endorse the intrauterine contraceptive device. These doctors raised three main objections to justify their stance. The first was that there were health risks associated with the Ōta ring that outweighed any benefits (e.g. Moriyama, 1939). Second, and similar to the argument deployed by the pro-life movement in the USA in the 1980s (Takeshita, 2011: 104–21), leading gynaecologists, such as Sawazaki Chiaki at the prestigious Department of Medicine of the Imperial University of Tokyo, claimed that rather than fulfilling its intended objective to prevent conception, the contraceptive actually induced abortions (Sawazaki, 1938). Third, eminent eugenicists in reproductive medicine, such as Hisomu Nagai and Yoshio Koya, considered unregulated fertility control to be a threat to eugenic principles; unchecked birth control could promote what they termed ‘reverse selection’ (‘gyaku tōta’), the expansion of groups with ‘inferior’ traits against those with ‘superior’ traits, which would inevitably ‘lower’ the quality of the Japanese population (Koya, 1941, Ōta, 1976, Suzuki, 1983). As these doctors were effective political lobbyists, their opposition to birth control was a critical factor in the reception of the Ōta ring.

Partly in response to these politically influential doctors, the Japanese Government passed legislation throughout the 1930s to eradicate birth control practices, which ultimately limited public access to the IUD. A regulation was issued in 1930 intending to limit the distribution of what were regarded as ‘harmful contraceptives’ (‘yūgai hiningu’), and in 1936, the IUD were defined as such. These regulations initially allowed the use of the IUD in clinical research, temporarily providing an opportunity for doctors such as Ōta to popularize the contraceptive (Ōta, 1974: 1–62, 107). However, the situation changed after Japan declared war on China in 1937. Thereafter, the Japanese Government adopted a pronatalist population policy in order to ensure a continuous supply of the so-called ‘human resource’ (‘jinteki shigen’). In 1941, the Cabinet passed a ‘Summary of Points on the Establishment of Population Policy’ (‘Jinkō Seisaku Kakuritsu Yōkō’), Item 4 of which recommended the banning of artificial contraception methods and induced abortion (Kubo, 1997: 62). From then onwards, as Ōta himself stated, ‘even research on the Ring was interrupted’ (Ōta, 1979: 238). The government campaign to tame the birth control movement, along with eugenisist notions present since the 1920s, were ultimately absorbed by this wartime pronatalist policy, preventing birth control campaigners from widely distributing contraceptives, including Ōta's IUD.

## The IUD in the early post-war birth control campaign

After the Second World War, there was a reverse in policy and the Japanese Government became among the first in the world to adopt the popularization of birth control as a national project (Kubo, 1997, Ogino, 2008a). This position was ratified at a meeting held on 26 October 1951, and in 1952, the Eugenic Protection Law of 1948 was amended to permit existing eugenic marriage consultation clinics to promote birth control. The Government also ordered local authorities to provide community-based ‘birth control field instructors’ (‘jutai chōsetsu jicchi shidōin’) to teach the public about birth control and distribute contraceptives at wholesale prices (Homei, 2016b, Obayashi, 1989).

This radical transformation in the Japanese Government's attitude to birth control derived from two circumstances emerging immediately after the Second World War. The first was rapid demographic change leading to escalating population figures. Between 1947 and 1949, Japan not only gained 8.06 million babies, but also approximately 7 million repatriates from former colonies and the warfront. From 71 million in October 1945, the population of Japan had increased by over 10 million by the early 1950s, and it was estimated that the figure would reach 100 million by 1962 (Ogino, 2008b: 142). Confronted with a surge in public debates on what was characterized as ‘overpopulation’ (‘kajō jinkō’) – deemed to exacerbate existing social problems such as a shortage of food and housing, unemployment, epidemics and vagrancy – the Government recognized the urgency of the situation (Zaidan Hōjin Jinkō Mondai Kenkyukai, 1946a, Zaidan Hōjin Jinkō Mondai Kenkyukai, 1946b). This concern was shared by Douglas McArthur, Supreme Commander of the Allied Powers' General Headquarters (SCAP-GHQ) under the Allied Occupation of Japan (1945–1952), who believed that rapid population growth could slow down the industrial reconstruction of Japan and therefore hamper progress towards national independence (Dinmore, 2006, O'Bryan, 2009). Anxiety surrounding population growth at SCAP-GHQ was a significant element behind the government-sanctioned birth control policy, despite McArthur officially maintaining a neutral stance on the matter (Oakley, 1978).

The second factor was a surge in abortion figures following the Eugenic Protection Law (EPL) of 1948. Derived from the wartime National Eugenic Law (established 1940), this was intended to ‘prevent the birth of inferior offspring from the eugenic point of view’ and ‘protect the health and life of mothers’. The EPL was borne from anxiety among health officials and policy intellectuals over racial degeneration, fuelled by the increasing visibility of street children, mixed-race children, sexualized ‘pan pan girls’, and others perceived to symbolize post-war social degeneration (Matsubara, 1998). Once enacted, however, the EPL had the unintended consequence of encouraging induced abortion. In particular, a 1949 amendment permitted women to seek abortion on economic grounds, which *de facto* legalized the practice (Kato, 2009, Norgren, 2001). Having witnessed the abortion rate skyrocket immediately after this amendment, eugenicists launched a campaign to replace abortion with contraception. Most notable among these was Yoshio Koya, Head of the National Institute of Public Health. As well as fearing that women were placing their health at risk by using abortion as a means of birth control (Koya, 1954), Koya also believed that this unchecked contraceptive practice might trigger the aforementioned ‘reverse selection’. To counter this, Koya recommended a checked birth control initiative comprising of the promotion of conception control to target carefully selected populations (Koya, 1952: 2). Koya managed to persuade the Minister of Health and Welfare, Hashimoto Ryōgo, to accept his proposal, and secured a place on the Japanese Population Problem Council (established in the Cabinet in 1949), which drafted a proposal. This became the basis for the Cabinet's recommendation of birth control in 1951. The lobbying by eugenicist Koya and his supporters to replace contraception with induced abortion had mobilized the Japanese Government to endorse birth control (Homei, 2016a).

Meanwhile, a changing political climate was providing opportunities for previously suppressed birth control advocates to resume activities. Significant figures included Katō Shizue, Margaret Sanger's Japanese confidante, Kitaoka Juitsu, and physicians Majima Yutaka and Fukuda Masako (Hopper, 1996, Matsubara, 1998, Ogino, 2008b, Sei to Sheishoku No Jinken Mondai Shiryō Shūsei, 2001, Sugita, 2013). Ōta also played a critical role. In November 1945, he organized the Birth Control Alliance in Kyoto, and extended his clinic to provide a birth control consultation service.

Riding on the increasing popularity of birth control, doctors created various IUDs during this period. In 1948, Ōta resumed production of his golden devices in Tokyo, and collected trial data by coordinating with doctors Kimura Hitoshi and Tanaka Kazuo, who ran small clinics in the city districts of Ikebukuro and Hatsudai, respectively (Ōta, 1974: 84). A former colleague of Ōta developed a commercially viable version of the ring, which he patented under the name of the ‘Yūsei ring’ (Eugenic ring) (Ōta, 1974: 84–86), and in 1956, Kamakura-based doctor, Kurokawa Kazuo published a review of the device (Ōta, 1974: 105–106).

This boom in production was accompanied by an increase in the availability of raw materials, which generated diversification in the composition of IUDs. Ōta himself developed various models under the name of the Ōta ring. Having previously utilized mainly natural materials such as gold, rubber, silk gut and hair, he began to experiment with artificial materials immediately after the war. Ōta first tried Sunplatina (‘Sanpura’), a domestically produced nickel-based alloy used in dentistry, and as synthetic fabrics began to be imported from the USA in the late 1940s, tested many of these materials, including nylon in 1949, vinyl in 1951, plus combinations of vinyl and nylon in 1951, and combinations of polyethylene and nylon in 1954, 1955 and 1960.

Along with changes in raw materials, the design of the Ōta ring also underwent various transformations. Having been either a simple, smooth-surfaced ball or ring before 1945, Ōta introduced notches to the outer part of the ring as elastic synthetic materials became available after the war. Continuing to experiment with various alternative designs thereafter, he finally settled on a version with nylon thread wrapped around a smooth-surfaced polyethylene ring with a polyethylene marble placed within the centre of the ring's inner circle and attached with three threads (Ōta, 1974: 100–102).

This diversification in design during the early post-war period derived from creators' concerns about technical issues specific to intrauterine devices. Ōta, for example, introduced notches to solve the persistent problem of expulsion in the existing model. However, after 1951, he was compelled to reconsider the new design. The model produced that year became so firmly lodged in the uterine wall that, after several months of insertion, it was impossible to remove; a chemical reaction inside the uterus was causing the cog-like outer feature to harden. This example illustrates how a designer's constant efforts to balance efficacy and health risks drove changes in intrauterine contraceptives, but these changes did not necessarily always lead to improved welfare for users.

Despite a boom in production, the IUD was marginalized within the state-endorsed birth control campaign for two main reasons. First, this method had already been excluded from the campaign's pilot studies, organized by Yoshio Koya throughout the 1950s to test the efficacy of a state-endorsed birth control programme (Homei, 2016b). Koya did not provide a clear reason for the exclusion of the IUD, but evidence suggests that it was because the contraceptive did not satisfy any of the three criteria he used, namely public popularity (e.g. condom), low-tech design (e.g. rhythm method), and that Koya himself could conduct a clinical trial on the product (e.g. the foam tablet, Sampoon) (Koya et al., 1958).

The second and more significant reason was the Japanese Government's continuing ban on the IUD. In August 1948, in part to tackle population growth, a reformed Pharmaceutical Law was issued to deregulate the ban on those contraceptives previously deemed ‘harmful’. Throughout May 1949, the Ministry of Health and Welfare issued a series of authorizations for the distribution of 26 birth control medicines (Kubo, 1997, Tama, 2016), including the contraceptive jelly brand Contra and tablets such as the Eizai Pharmaceutical Company's Sampoon. Yet prohibition of the IUD remained, with prefectural mayors receiving official notification specifically clarifying the position of the Japanese Government (Ōta, 1974: 117). Thus, when Ōta applied to the Ministry of Health and Welfare after 1948 for approval of his ring, the application was rejected.

While this reaction frustrated Ōta, from the point of view of health officials attempting to weigh the contraceptive's efficacy against its health risks, a cautious approach simply made sense. The main concern was that technology ‘foreign’ to the body might produce adverse reactions once inserted in the uterus, causing disorders such as necrosis, inflammation and infection. After careful consideration, the Ministry of Health and Welfare ultimately concluded that potential risks outweighed any benefits that the IUD could provide (Ōta, 1974: 118). However, ministry officials had not entirely dismissed the potential of the device to curb population growth, and allowed doctors to conduct clinical trials. The Government's cautious approach was therefore based on balancing the contraceptive's efficacy with its safety; a calculation that established the contraceptive's marginalization in the government-endorsed birth control campaign.

In such a favourable environment for birth control, the IUD could theoretically have been popularized widely during the early post-war period. The emerging discourse on the need to restrict population growth helped to generate birth control policies and liberated hitherto suppressed birth control activism. Furthermore, the availability of a wider range of materials triggered innovation and a diversification of contraceptive options. Despite this, the IUD continued to appear only in a clinical setting. This marginal position was attributable to the interaction between a government relying on Koya's pilot project for justification of its birth control policy, and Koya's scientific study emerging out of the government campaign. As neither the Japanese Government nor Koya valued the method, the IUD was absent from the state-endorsed birth control programme, and was only promoted sporadically in private clinics via clinical trials reliant on personal networks.

In the process of marginalizing the contraceptive within official domains, government officials highlighted potential health risks, which they argued outweighed efficacy. Considerations of efficacy and risk, however, were not the only reasons for this marginalization. As I will illustrate in the following section, the pre-existing discourse relating to the device – the so-called ‘ring dispute’ – fostered by the relationship between prominent medical figures and central government, also contributed to suppressing dissemination of the IUD in the early post-war period.

## The ‘ring dispute’

In part because of the surge in innovation described above, the 1950s witnessed the publication of an increasing number of clinical trial reports (Doi, 1954, Hashimoto and Nakamura, 1952, Ishihama, 1954b, Ishihama, 1957, Mitsui, 1956, Nakamura et al., 1959, Yasuda and Sasai, 1954, Yoshida, 1957).

Having been conducting clinical trials to establish his contraceptive's efficacy prior to the period, Ōta was at the forefront of this trend. When his application for authorization of the Ōta ring was rejected, despite the Pharmaceutical Law reforms, he decided to put more energy into clinical trials. To start with, at a meeting of the Kinki Society of Gynaecology held in Takarazuka in November 1949, Ōta reported on 39 clinical trial cases using the Ōta ring based on data collected during the war, and published the presentation in the journal *Progress in Obstetric-Gynecology* in 1950 (Ōta, 1950). This seemingly innocuous report, which demonstrated that 34 out of 39 women had shown no sign of complications and five out of 39 who had the device removed had successfully given birth up to three times, led to a series of academic debates in the early 1950s that Ōta retrospectively termed the ‘ring dispute’ (‘ringu ronsō’) (Ōta, 1974: 87–99).

The ‘ring dispute’ unfolded within a community of doctors specializing in obstetrics, gynaecology and paediatrics, with battle lines drawn between proponents of the device, largely clinicians, and those against the use of the IUD, who were mostly academic doctors at elite institutions with government connections. At the heart of the dispute lay the issue of the efficacy of the IUD for fertility control, and how the participants interpreted clinical trial data presented during the debate. Advocates of the IUD repeatedly emphasized efficacy by focusing on success rates, while their opponents pointed out the potential health risks revealed in the data, such as cases of bleeding and expulsion (Ishikawa, 1952a, Ishikawa, 1952b, Ishikawa, 1952c).

The dispute reached a climax on 31 January 1952 at the Tokyo Local Meeting of the Japan Society of Obstetrics and Gynaecology. In Ōta's presence, the doyen of obstetric-gynaecology, Andō Kakuichi, from the prestigious private Keio University Medical School, harshly criticized the Ōta ring. Despite disagreeing with the claim by Sawazaki that the ring induced abortions, and accepting that the IUD did indeed prevent conception *in utero*, Andō essentially agreed with Sawazaki that the accompanying health risks should be avoided by adopting other, simpler and easily available techniques, such as barrier methods. In response, Ōta accused Andō of being too theoretical and demanded he provide supporting data. After a long, heated discussion, a consensus could not be reached, and Andō concluded the meeting by reminding the audience that obstetrician-gynaecologists should never recommend any contraceptive method that was not proven to be 100% safe (Ishikawa, 1952a: 276).

Participants in the ‘ring debate’ were characterized by their proximity to political decision-making. The main constituents who opposed the IUD were doctors at elite academic institutions, heavily involved in the process of establishing the Government's policies on reproduction. In contrast, many IUD proponents were rank-and-file clinicians or maverick doctors such as Ōta, who, while generally excluded from policy procedure, had significantly more contact with women actually using the device. Furthermore, while the majority of the opposing camp enjoyed physical proximity to central government, many – such as Yagi Hideo, who lived a long way from the capital – were more prone to political isolation.

This sociopolitical position of the debate's participants, which translated into accessibility to political power, appears to have contributed to moulding official opinion on the IUD. The government consultation on the Ōta ring that started in September 1953 illustrates this point. In response to Ōta's formal appeal, the Ministry of Health and Welfare set up a Special Committee on Intrauterine Contraceptive Devices (Ōta, 1974: 117–118), but did not invite Ōta or any other clinician in the pro-ring camp to take part. Only noted figures were appointed committee members, including the aforementioned Sawazaki and the anti-ring paediatrician, Moriyama Yutaka. In 1959, after a clinical trial of 14,065 nylon rings and 2285 metal rings, the committee unanimously concluded that governmental approval of the device would be premature, and that controls preventing use of the IUD outside clinical trials should be strengthened (Ōta, 1974: 118–120). Throughout the rest of the 1950s, the Japanese Government maintained this conservative stance towards the IUD; a stance informed less by the demographic imperative, its own birth control policy or clinical trial data than by the position of doctors in a larger political context.

## Emerging interest in the IUD and the transnational movement during the 1960s

By the end of the 1960s, in a complete *volte-face*, the Japanese Government was enthusiastically embracing use of the IUD, to the extent that in 1974, the Ministry of Health and Welfare permitted production of the Ōta and Yūsei rings as medical contraceptives. The Lippes loop, originally developed in the USA, also became available to the public in 1976. Also that year, Fuji Latex Co. Ltd. – a Japanese corporation known for producing condoms – began to produce FD-1, its own IUD, which resembled a fishbone. By 1978, a total of 2,166,250 IUDs had been produced in Japan, of which approximately 80%, or 1,730,570, were for domestic use (Ishihama, 1980: 664).

Why did the Japanese Government, hitherto so reluctant, finally give the IUD the green light? To answer, I argue that the Government's interest in this contraceptive method, which increased rapidly through the 1960s, was shaped by both domestic and transnational contexts.

Within Japan, a sizable number of women had been exposed to various forms of IUD by the 1960s via clinical research. Influential institutions also began to participate in clinical research on the contraceptive in that decade, resulting in the distribution of over 40,000 Ōta rings (Kazoku Keikaku Renmei, 1969, Ōta, 1974). By the late 1960s, the IUD had become a recognized contraceptive technology in women's everyday lives, with magazines targeting married women characterizing the IUD as the contraceptive of the future (Dōshitara Yoi? Korekara No Hinin, 1969, Shinkon Seikatsu Wo Yutaka Ni Suru Ai No Hininhō, 1969, Watashitachi Wa ‘Ringu’ de Hinin Ni Seikō Shita, 1967). This public visibility was an important background to the Government's shift in attitude towards the IUD.

However, in addition to the domestic factor, I argue that the burgeoning transnational movement to curb population expansion in those regions deemed the ‘Third World’ ultimately swayed the Government's stance. Spurred by the emerging theory of socio-economic development that equated overpopulation with poverty, and by Cold War geopolitics, in which the USA came to consider development aids as tools of what Christina Klein termed the ‘global imaginary of containment’ (Klein, 2003: 23), US institutions promoted family planning in the non-aligned or ‘free world’ developing countries as part of its development aid programmes from the mid-1960s (Latham, 2011, McCann, 2016 Sharpless, 1995, Sharpless, 1997). This trend interacted directly with the transnational population control movement, spurring global dissemination of the IUD. The Population Council, a New York-based philanthropic organization that had funded research on the IUD since the 1950s, began to actively promote the Lippes loop and Margulies spiral to Third World family planning initiatives by collaborating with international family planning agencies (Donaldson, 1990, Takeshita, 2011). Consequently, the IUD became one of the most common methods of contraception deployed in the transnational family planning initiatives of the 1960s. Within Asia, the IUD was embraced most enthusiastically by South Korea and Taiwan, who developed their own initiatives in cooperation with the Population Council (DiMoia, 2008, Donaldson, 1990, Huang, 2009, Huang, 2016, Kuo, 2002).

The rapid global circulation of the IUD, and its popularity in Japan's neighbouring countries in particular, swayed bureaucratic attitudes to such an extent that the possibility of authorizing the IUD was reconsidered. In August 1964, the Ministry of Health and Welfare requested advice from the Japan Society of Obstetrics and Gynaecology regarding whether or not the IUD was safe and could be recommended to the public (Ōta, 1974: 114). Three years later, in September 1967, the Ministry of Health and Welfare repeated the inquiry, but this time not only to the Japan Society of Obstetrics and Gynaecology but also to the Japan Family Planning Federation and the Japan Association for Maternal Welfare, historically the most influential medical lobbying group for induced abortion (Ōta, 1974: 128). International developments had exhorted the Japanese Government to at least consider revising its view on the device.

However, it must be stated categorically that the transnational movement was more than simply a phenomenon of ‘gaiatsu’, or ‘foreign pressure’, which Norgren has defined as a ‘blunt tool’ which ‘small, political weak groups can use effectively to turn attention to [a given] issue’ (Norgren, 1998: 76). Instead, it was a constitutive element in domestic policy. Furthermore, what enabled this constitutive relationship between the transnational movement and domestic policy was the active engagement of Japanese doctors with the transnational movement.

What is particularly fascinating about this aspect of IUD history is that those Japanese doctors who participated in the transnational dialogue about the IUD were not the elite. In a shift from the previous era, when those with the most prestigious positions shaped government policy, the most significant Japanese actors for the development of the IUD from an international perspective were not Sawazaki and his noted colleagues, but Ōta and a seemingly unassuming doctor, Ishihama Astumi (Ishihama, 1976). Ōta became renowned throughout South-east Asia during the 1960s, especially in Malaysia, where the Government enthusiastically adopted family planning initiatives to boost the country's socio-economic development (Ōta, 1975). In turn, Ishihama established himself as the chief promoter of Ōta rings among the international community of IUD researchers (Margulies, 1975: 664).

Ishihama's career had humble origins. Although he was a graduate of one of the prestigious imperial universities, it was based on the island of Kyushu, a long distance from Tokyo. Furthermore, he spent most of his early career in the remote Iwate University. However, this peripheral position enabled Ishihama to establish his career with the contentious Ōta ring. Ishihama first considered recommending the Ōta ring after suffering exhaustion from repeatedly performing mid- to late-term abortions on female repatriates in the provincial Prefecture of Fukuoka. This was a covert operation organized by the Ministry of Health and Welfare to prevent the birth of children conceived potentially as a result of rape or other incidents that occurred during the repatriation process (Ishihama, 1998, Ishihama, 2004). Ishihama then secured a position at Iwate University and on discovering the insufficient size of his research budget, decided to conduct a clinical trial of the Ōta ring with local women, partly because he believed this could be done fairly inexpensively (Ishihama, 2004: 23–37). Ishihama contacted Ōta and, with Ōta's donation of his rings, was able to conduct a clinical trial with 600 local women (Ishihama, 1954a), and later a physiological examination of 20,000 women.

On the recommendation of Shinoda Tadasu, his senior colleague and Dean of the Medical School at Iwate University, Ishihama published the results of the latter study in both English and Japanese (Ishihama, 1957, Ishihama, 1959, Ishihama, 2004: 40). Published in the *Yokohama Journal of Medicine*, the English version eventually came to the notice of Alan Guttmacher at the Population Council, the driving force behind global promotion of the IUD, via Christopher Tieze and Clarence J. Gamble, who were both involved in the development and assessment of contraceptive methods deployed internationally (Ishihama, 2004: 58). Guttmacher invited Ishihama to the First International Conference on the IUD held in New York in 1962, at which the term ‘IUD’ was coined (Ōta, 1974: 60). Due to Ishikawa's presentation, the Ōta ring became recognized as a viable contraceptive method. At the 1967 International Family Planning Conference in Chile, Guttmacher introduced the Ōta ring to the public and even referred to Ōta as the ‘Father of the IUD’ (Ōta, 1979: 240).

The international recognition of Ishihama and Ōta significantly altered the dynamics within the community of doctors responsible for government policy. Ishihama, the only Japanese doctor invited to speak at the first-ever international conference on the IUD, rapidly established himself in the international arena during the 1960s (Ishihama, 2004: 80). Thus, those medics closest to policy making welcomed Ishihama into their clique. Between 1965 and 1968, Ishihama was invited to speak about the IUD at meetings of the Japan Society for Infertility in Kanazawa and the Japan Society of Obstetrics and Gynaecology in Yonago (Ishihama, 2004, Ōta, 1974). Ishihama's presence in the body of top medical researchers gradually helped tilt opinion towards favouring the IUD.

Nevertheless, Ishihama could not alone have swayed the opinion of his anti-IUD colleagues. Throughout the 1960s, these doctors also began to engage with the transnational movement, which urged them to revise their conservative views on the IUD. For instance, Takashi Kobayashi, President of the Japan Society of Obstetrics and Gynaecology, immediately began to support IUD use after accepting an invitation from the Population Council and attending the Second International Conference on the IUD in 1964 (Ōta, 1974: 98). Kobayashi's new attitude reflected the general mood within the community of hitherto anti-IUD doctors. In April 1968, to prepare an informed response to the Ministry of Health and Welfare's inquiry on the question of the government approval for the IUD, the Japan Society of Obstetrics and Gynaecology set up the IUD Investigation Committee (‘IUD Chōsakai’), which spent a total of 4 years investigating use of the IUD and the associated health risks (Matsumoto, 1972, Mizuno, 1970, Mizuno, 1971). The proposal submitted by the IUD Investigation Committee in March 1971, which generally cast a positive view, paved the way for governmental authorization of the IUD in 1974.

Additionally, Koya, a renowned figure in international birth control since the 1950s, swiftly revised his view as the role of the IUD in transnational population control campaigns expanded (Homei, 2016c). The direct trigger came from a lecture Guttmacher delivered in Japan on his return from the 1965 IPPF West Pacific Region meeting held in South Korea. In the lecture, which Koya chaired, Guttmacher implicitly criticized the Japanese Government and doctors that still ‘adhered to the traditional and classic contraceptive methods and do not [encourage people to] use the oral contraceptive and IUD whose efficacy is almost 100%’ (Koya, 1969). After the event, Koya organized a subcommittee within the Medical Committee of the Japan Family Planning Federation, and commissioned a study on IUD efficacy. The result, published in 1969, provided an informed view on the contraceptive and became a driving force towards government approval (Kazoku Keikaku Renmei, 1969, Kubo et al., 1969). As Koya was a prominent figure whose opinions on birth control had been respected by the Ministry of Health and Welfare even before the war, his support for the IUD was also critical.

As this case illustrates, the transnational context of the 1960s played a pivotal role in the Japanese Government's eventual authorization of the sale and distribution of the IUD in 1974. Guttmacher's favourable opinion of the IUD, perceived as representing a voice of the translational population control movement, eventually urged elite medical circles and government health officials to revise their negative attitude to the contraceptive. Furthermore, a small number of Japanese doctors, such as Ishihama, changed the course of domestic policy making by being firmly embedded in the transnational movement with his internationally recognized medical research. The eventual official approval of the IUD was thus catalysed by those transnational elements which, far from being simply an external force imposed on national trends, helped erode the conservative attitude to the IUD from within Japan.

## Conclusion

So, why did the Japanese Government take so long to approve the intrauterine contraceptive method? To respond to this question, I have examined how medical doctors participated in the process of establishing state birth control policy, and played a significant role in the decision-making process in response to the assumption that their expert knowledge would provide insight.

However, the ‘ring dispute’ demonstrated that expert knowledge was not influential on its own; proximity to political power was the crucial factor determining which scientific knowledge was deemed more credible. Although activist doctors, clinicians and provincial university doctors used scientific data to show the efficacy of the IUD, the anti-ring faction's conservative view and its emphasis on health risks struck a chord with the Government to a far greater extent than the pro-ring faction's allegedly scientifically-proven argument. The fact that health officials, who were compelled to consider health risks when assessing contraceptives, already shared the stance of the anti-ring doctors, enabled this view to pervade government policy. However, as has been shown above, anti-ring doctors were also better able to shape government decision-making due to their close proximity to political power. Consequently, at least during the 1950s, the Japanese Government maintained a cautious approach to this contraceptive device.

During the 1960s, however, government policy changed dramatically and swiftly. This change in attitude corresponded with shifting dynamics within the body of doctors exerting political influence, and was partly generated by the boost to IUD distribution provided by the transnational population control movement, particularly in neighbouring societies within Asia. Crucially, this transnational trend was also firmly integrated in the domestic policy-making process. Those doctors who rose from relative obscurity to become major players in domestic reproductive politics, did so precisely because international audiences deemed them representative of the Japanese community of birth control researchers, and actively engaged with them in the global circulation of the IUD. Their scientific activities connected national and transnational arenas, and eventually superseded the previous approach, leading to authorization of the sale and distribution of the IUD in 1974.

In answer to this paper's principal question, then, I argue that the specific ways in which hierarchies among doctors – informed by their sociopolitical positions – shaped official evaluations of biomedical arguments, was a major factor behind the relatively long time it took the Japanese Government to approve use of the IUD. However, I also note that interactions between transnational and domestic reproductive politics in the 1960s, mobilized by a small number of Japanese doctors, consequently impelled the Government to approve the IUD not a great deal later than other countries in East Asia.
